# Dendritic Cells in Human *Pneumovirus* and *Metapneumovirus* Infections

**DOI:** 10.3390/v5061553

**Published:** 2013-06-20

**Authors:** Antonieta Guerrero-Plata

**Affiliations:** 1 Department of Pathobiological Sciences, Louisiana State University, Baton Rouge, LA 70803, USA; E-Mail: aguerrp@lsu.edu; Tel.: +1-225-578-9678; Fax: +1-225-578-9701; 2 Center for Experimental Infectious Disease Research, Louisiana State University, Baton Rouge, LA 70803, USA

**Keywords:** dendritic cells, human metapneumovirus, respiratory syncytial virus, lung, paramyxovirus

## Abstract

Lung dendritic cells (DC) play a fundamental role in sensing invading pathogens, as well as in the control of tolerogenic responses in the respiratory tract. Their strategic localization at the site of pathogen entry makes them particularly susceptible to initial viral invasion. Human respiratory syncytial virus (hRSV) and human metapneumovirus (hMPV) belong to the *Paramyxoviridae* family, within the *Pneumovirus* and *Metapneumovirus* genera, respectively. hRSV and hMPV are significant human respiratory pathogens that cause similar clinical manifestations and affect many of the same subpopulations. However, they differentially activate the host immune response, including DC, which represents a fundamental link between the innate and adaptive immune response. In this review, the role of DC in the immune response against hRSV and hMPV infections, as well as the inhibitory effects of these paramyxoviruses on the DC immunity will be discussed.

## 1. Introduction

**Figure 1:**
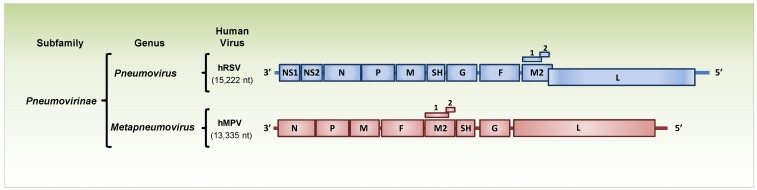
Schematic representation of human *Pneumovirus* and *Metapneumovirus*. Gene maps and encoded proteins of members of the subfamily Pneumovirinae: human respiratory syncytial virus (hRSV) and human metapneumovirus (hMPV), which belong to the genera *Pneumovirus* and *Metapneumovirus*, respectively. Genes are represented as boxes with the corresponding encoded protein.

Human respiratory syncytial virus (hRSV) and human metapneumovirus (hMPV) are classified within the *Paramyxoviridae* family, *Pneumovirinae* subfamily, which is divided into the *Pneumovirus* and the *Metapneumovirus* genera. Both viruses belong to the order Mononegavirales and contain a nonsegmented, negative-sense RNA with genomic organization, which is similar, but not identical [1,2,3,4]. Metapneumoviruses lack the nonstructural proteins NS1 and NS2, and the gene order is different from that of pneumoviruses. hRSV is the type species of the *Pneumovirus* genus, while (based on the biological properties and genomic sequence) hMPV has been assigned to the *Metapneumovirus* genus. hRSV encodes 11 proteins (nonstructural (NS) protein1, NS2, nucleocapsid (N), phosphoprotein (P), matrix (M)1, small hydrophobic (SH), attachment (G), fusion (F), M2-1, M2-2 and polymerase (L), Figure 1), while hMPV encodes nine proteins (N, P, M1, F, M2-1, M2-2, SH, G and L, Figure 1). 

hRSV was first isolated in 1956 from an infected chimpanzee [5] and represents the most important cause of bronchiolitis and pneumonia in infants and young children worldwide. More than 95% of children are infected with hRSV by two years of age [1]. hMPV was first identified in 2001 following its isolation from infants and children with lower respiratory tract infections (LTRI) of unknown etiology [2]. Serologic evidence indicated that hMPV seropositivity is almost universal by the age of five years. The clinical manifestations of hMPV infection in young children are indistinguishable from those of hRSV infection. LRTI associated with hMPV in infants and young children is a frequent cause of hospitalization. Several studies indicate that hMPV likely accounts for 5% to 15% of LRTI hospitalizations in infants and young children and is second only to hRSV as a cause of bronchiolitis in early childhood [6,7,8,9,10]. Both viruses can be transmitted mainly by large droplets from infected individuals [11,12], and the incubation period could last between three and seven days for hRSV and four to six days for hMPV [13]. hRSV and hMPV are present year-round, but their incidence increases in the fall, peaks in the winter and goes down in early spring [1,14,15]. Host risk factors for these infections include premature birth, congenital heart disease, immunodeficiency, elderly individuals, gender and Down’s syndrome, among others [16,17,18]. Adults and older children are commonly reinfected without complications by these paramyxoviruses, because the natural infection does not induce lifelong immunological protection. However, it is in infants, elderly and immunosuppressed individuals that severe disease can result [19,20,21]. Currently, there are no vaccines available against hRSV or hMPV. However, infected individuals can be treated with ribavirin and immunoglobulins [22].

Knowledge of the critical aspects of the host immune response to these infections has been crucial to understanding the pathology associated with hRSV and hMPV infections. In that regard, dendritic cells (DC) play a pivotal role in shaping antiviral immune response in the respiratory tract, as they represent the perfect link between innate and adaptive immune response [23,24,25]. Although the mechanisms underlying the activation of these cells by paramyxovirus infections still is largely unknown, substantial progress towards our understanding of the DC response to hRSV has been made, and the role of these cells in hMPV infection has also been explored.

## 2. Dendritic Cells in the Respiratory Tract

Dendritic cells are professional antigen-presenting cells within the immune system. They arise from both myeloid and lymphoid progenitors within the bone marrow and are widely distributed (as immature DC) into both lymphoid and nonlymphoid tissues [26,27,28]. Respiratory tract dendritic cells are present within airway epithelium, submucosa and associated lung parenchymal tissue under resting conditions [29]. In the absence of inflammation, lung DC are present at an average density of several hundred cells per square millimeter in the large airways, decreasing to less than a hundred DC per square millimeter within smaller intrapulmonary airways [30]. Pulmonary DC have a rapid turnover, with a half-life of ≤2 days [30,31]. Their strategic localization at the site of pathogen entry makes them particularly susceptible to initial viral invasion. After detection, uptake and degradation of viruses, DC initiate immune responses via the secretion of interferon, chemokines and proinflammatory cytokines, as well as the upregulation of a variety of costimulatory molecules and receptors, a process globally known as cell maturation. After maturation, DC efficiently present antigens and initiate adaptive immune response by migrating into lymph nodes (LN) to activate the virus-specific T-cell response [32]. 

## 3. Activation and Inhibition of DC Infected with hRSV and hMPV *in Vitro*

### 3.1. DC Maturation and Cytokine Production

DC maturation is necessary for the transition from innate to adaptive immunity. In human monocyte-derived DC (moDC), hRSV and hMPV are able to induce cell maturation, indicated by an upregulation of MHC class I and class II molecules, as well as the overexpression of CD80, CD86, CD38 and CD83 [33,34,35,36,37]. Cytokine production by these cells is differentially induced by hRSV and hMPV, hRSV being a more potent inducer of IL-10, TNF-α, IL-1β and IL-12p70, while hMPV induces a more robust response of type I IFN than hRSV. IFN induction is dependent of viral replication, as UV-inactivated virus failed to induce IFN in hRSV- or hMPV-infected cells [34,35,36,38]. The response of human primary blood myeloid DC to hRSV has also been characterized, and it resembles that of moDC [39]. hRSV also induces maturation in mouse DC. Bone marrow-derived DC (BMDC) exhibited an increased expression of CD40, CD80, CD86, MHC class I and class II and higher production of IL-6, IL-10 and IL-12p70. Consistent with reports in human cells, hRSV replication was required also for DC maturation [40]. However, DC are not highly permissive to hRSV or hMPV infections, regardless of their source (PBMCs, cord-blood or primary DC), as demonstrated by the low percentage (4% to 25%) of antigen positive cells by flow cytometry analysis [33,34,35,36,37,39,41]. This suggests a critical role of the virus-induced cytokines, such as type I IFN, during the maturation process, as reported in BMDC [42]. Infection of plasmacytoid *dendritic cells* (pDC) by hRSV and hMPV induce differential expression of cytokines and chemokines. As in moDC, hRSV is a more potent inducer of IL-6, IL-10, GM-CSF, TNF-α, IL-1β, IL-12p70 and G-CSF than hMPV. However, both viruses induce a similar response of IFN-α release in infected pDC [33]. 

In understanding the mechanisms of activation and maturation by hRSV, Munir* et al.* [43] have reported that the NS proteins of hRSV suppress the expression of costimulatory molecules, as well as the secretion of cytokines and chemokines in human moDC in a type I IFN-dependent process. The inhibitory effect was mediated mostly by NS1 protein, but was enhanced by the combined deletion of NS2 in the same recombinant hRSV virus. More recently, Johnson* et al.* [44] reported that the interaction of hRSV G protein with the DC/L-SIGN (C-type lectins commonly found in DC) inhibits maturation of primary human DC. They found that when this interaction was neutralized with specific antibodies, RSV-infected myeloid DC and pDC increased both maturation and cytokine/chemokine production [44]. The role of cellular mechanisms critical for DC function has also been explored. Morris* et al.* [45] investigated the role of autophagy, a cellular mechanism that involves cell degradation of unnecessary or dysfunctional cellular components [46]. They found that cytokine production and the expression of surface markers (MHC class II, CD40, CD80 and CD86) was inhibited when autophagy was blocked in hRSV-infected BMDC, indicating that autophagy is a critical cellular process for DC maturation during hRSV infection [45]. 

### 3.2. Activation and Inhibition of IFN Responses

Interferons (IFNs) are a heterogeneous family of cytokines with demonstrated antiviral, antitumor and immunomodulatory activities. The IFN family includes type I (IFN-α, -β, -ε, -κ and -ω), type II (IFN-γ) and type III (IFN-λ1, -λ2 and -λ3) IFNs [47,48]. Local production of IFNs plays an important defensive role in many respiratory virus infections by limiting viral replication until virus-specific host defense mechanisms develop [49]. It is known that hRSV and hMPV are able to induce and inhibit the IFN response in human and mouse DC. Moreover, they use different molecular mechanisms depending of the DC subset infected (Table 1). The induction of IFN production by RNA viruses is triggered by the activation by several pattern recognition receptors that recognize different viral components. Among them, the cytosolic RNA helicases, retinoic acid-inducible gene (RIG-I) and melanoma differentiation-associated gene 5 (MDA5) play a role in recognizing short or long dsRNA, respectively [50]. Other receptors include Toll-like receptors (TLR3 and TLR7) found in the endosomal compartment, which recognize dsRNA and ssRNA, respectively [51]. 

As previously reported, both hRSV and hMPV induce a robust response of IFN-α in human pDC, and this production is dependent on viral replication [33,38,52,53,54]. Using splenic mouse pDC, it has been further demonstrated that IFN production by hMPV is dependent on TLR7 expression [54]. Unlike in pDC, moDC produce IFN-α mostly after infection with hMPV, not with hRSV [33,38]. Using human moDC, previous results from my laboratory have demonstrated that the cytosolic helicase, MDA5, contributes to the production of type I and type III IFNs after hMPV infection. Those observations were further confirmed in an* in vivo* model of infection using MDA5^−/−^ mice [55]. Activation of TLR4 has also been reported to contribute to the production of IFN-β in moDC after hMPV infection [56].

Although the mechanisms underlying the differential activation of IFN response by hRSV and hMPV in moDC have not been fully elucidated, some experimental evidence indicates the involvement of several viral proteins. Munir* et al.* [43] demonstrated that the hRSV NS1 and, to a lesser extent, NS2 protein suppress the expression of IFN-α/β by using recombinant RSVs bearing deletions of the NS1 and/or NS2 protein [43]. In the case of hMPV, it is the expression of the protein G that is responsible for the reduced production of IFN-α/β in infected moDC [56]. 

hRSV and hMPV infections are also known to subvert the immune responses by interfering with DC functions. The modulating mechanisms of DC immunity by these viruses have been investigated in several* in vitro* systems, including human DC. It has been shown that hRSV and hMPV inhibit the production of IFN-α in human pDC in response to TLR7 and TL9 agonists [33,52] and in moDC after TLR3 activation [33]. In addition, the soluble form of the G protein of hRSV and hMPV has also been reported to block the IFN response after TLR activation in moDC [56,57]. 

Overall, these data indicate that hRSV and hMPV activate and inhibit the IFN responses in DC, most possibly through different mechanisms. This critical knowledge contributes to our understanding of the molecular mechanisms of hRSV and hMPV immunopathogenesis and may help to explain the lack of protective immunity after natural infection and the multiple reinfections by these viruses.

**Table 1:** Activation and inhibition of IFN responses by human respiratory syncytial virus (hRSV) and human metapneumovirus (hMPV) in dendritic cells (DC). pDC, plasmacytoid *dendritic cells*; moDC, monocyte-derived DC.

**Virus**	**Effect**	**DC Subset**	**References**
hRSV	Induce IFN-α	pDC	[33,38,52,53]
hRSV	Blocks TLR7 and TLR9 activation	pDC	[33,52]
hRSV	Blocks TLR3 activation	moDC	[33]
hRSV NS1 and NS2	Suppress IFN-α/β production	moDC	[43]
hRSV soluble G	Blocks TLR3 and TLR4 activation	moDC	[57]
hMPV	Induce IFN-α	pDC	[33,54]
hMPV	Induce IFN-α, β, λ	moDC	[33,55]
hMPV	Activates MDA5	moDC	[55]
hMPV	Activates TLR4	moDC	[56]
hMPV	Activates TLR7	pDC	[54]
hMPV	Blocks TLR7 activation	pDC	[33]
hMPV	Blocks TLR3 activation	moDC	[33]
hMPV G	Suppresses IFN-α/β production	moDC	[56]

### 3.3. Regulation of T-Cell Responses

Another aspect of the biology of hRSV and hMPV is that they reinfect throughout life, suggesting incomplete or transient immunity [19,58,59,60]. Several pieces of evidence indicate that hRSV and hMPV interact with dendritic cells and that the primary T-cell response to these viruses is altered significantly by this interaction. Human DC infected with hRSV have shown a severely impaired capacity to stimulate naive CD4^+^ T-cell proliferation [33,36,61]. The possible mechanisms of this inhibition has been attributed to soluble factors in the supernatant of hRSV-infected dendritic cells [36], as well as to direct contact with RSV-infected cells to inhibit proliferation of T-cells [52,61]. In fact, *Gonzalez et al.* [40] demonstrated, in bone marrow-derived DC (BMDC), that immunological synapse assembly between hRSV-infected DC and T-cells was impaired, supporting the notion that contact is necessary for the inhibition of T-cell activation by hRSV infection [40]. Inhibition of T-cell proliferation by hMPV-infected DC, however, has also been attributed to soluble factors secreted by hMPV-infected DC, but not by interference with DC-T-cell immunological synapse formation [62]. Others have not observed this inhibitory effect using hRSV-infected cord blood-derived DC or moDC cultured with naive T-cells and superantigens [37,63]. Whether this discrepancy in the activation of the T-cells by hRSV-infected cells is related to the different experimental conditions remains to be determined. Further characterization of the interaction between T-cells and hRSV- and hMPV-infected DC is warranted.

## 4. Response of Human Dendritic Cells to hRSV Infection *in Vivo*

Current characterization of human lung DC populations includes three types of DC: two myeloid DC (mDC) in the lung parenchyma (mDC1 (BDCA-1^+^/HLA-DR^+^) and mDC2 (CD11c^+^/BDCA-3^+^)) and one pDC subset (CD11c^−^/BDCA-2^+^/CD123^+^/CD14^−^/HLA-DR^+^) [64,65,66]. Additional reports have confirmed the presence of these lung DC subsets in human bronchoalveolar lavage samples [67,68]. However, further characterization of human lung DC populations, under the steady state and in response to stimulus, is needed in order to define the composition of human DC in the lung. 

There have been a limited number of studies focused on the DC response to hRSV or hMPV infections in humans. In fact, the response of pulmonary DC in hMPV-infected individuals has not yet been reported. However, despite the human sample limitations, analyses of nasal washes from young children with acute hRSV infection have revealed that hRSV attracts both mDC and pDC to the site of viral entry. The number of pDC, and, to a lesser extent, that of mDC, positively correlates with the viral load in the infected individuals [69]. However, hRSV recruits DC to a lesser extent relative to other relevant respiratory viruses, such as influenza virus [70]. On the other hand, the numbers of mDC and pDC decreased in peripheral blood, suggesting that the increase of mDC and pDC in nasal mucosa results from their migration from the blood [69,70]. In line with those data, Silver E.* et al.* [71] has found that the lower levels of pDC in peripheral blood have been associated with asthma after severe hRSV bronchiolitis [71]. Overall, these data indicate a relevant role of DC in hRSV infection in humans. However, the better understanding of the response of DC to hRSV or hMPV has been revealed from* in vitro* experiments using human and mouse cells and from experimental animal models, specifically, the mouse model. 

## 5. Response of Pulmonary DC in Experimental Mouse Models after hRSV and hMPV Infection

**Figure 2:**
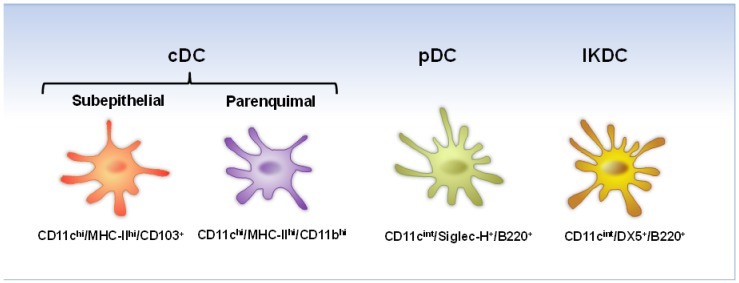
Mouse lung DC activated during hRSV and hMPV infection.

Because the study of the human DC at multiple stages of respiratory viral infections is technically and ethically difficult, the experimental mouse model has provided an excellent opportunity to investigate the response of DC* in vivo*. To date, there have been at least three major subsets of murine lung DC described. These include plasmacytoid DC (pDC), the myeloid DC (also known as conventional DC (cDC)) and the interferon-producing killer dendritic cells (IKDC) (Figure 2). All three DC populations have been reported to participate in the innate and adaptive immune response to hRSV and hMPV infections, indicating their critical role in the antiviral immunity to these viruses.

pDC are identified by the expression of CD11c^int^/B220^+^/Gr-1^+^/Siglec-H^+^/mPDCA1^+^. They are best known for secreting large amounts of type I interferons (IFN) in response to viral infections [72]. Type I IFN confer resistance to viral infections and promote apoptosis of virally infected cells [73]. Also, type I IFN promote myeloid DC, B-cells, T-cells and natural killer (NK) cell functions [74,75]. Therefore, pDC regulate both innate and adaptive immune response in viral infections. pDC can also present endogenous viral antigens in their activated state to CD4^+^ T-cells, but they are less efficient antigen-presenting cells, as compared to cDC [76]. Additionally, pDC are able to produce a variety of cytokines and chemokines that are important for the activation and trafficking of CD4^+^ and CD8^+^ T-cells to the site of infection [77,78]. 

cDC are identified as major histocompatibility complex class II (MHC-II)^hi^/CD11c^hi^. They are considered the main antigen-presenting cells of the immune system [26,79]. In mice, lung cDC comprise two major cDC subpopulations based on the expression of the integrin marker, CD103, and myeloid marker, CD11b, to give place to MHC-II^hi^/CD11c^hi^/CD11b^−^/CD103^+^ in the intraepithelial network and the parenchymal MHC-II^hi^/CD11c^hi^/CD11b^+^/CD103^−^ DC [79]. These two cDC subsets differ in their relative abilities to prime CD4^+^ and CD8^+^ T-cells [80], produce proinflammatory cytokines and generate Foxp3-mediated regulatory function of naive T-cells [81,82]. CD103 (α_E_) is the α-chain of the α_E_β_7_ integrin that mediates human and mouse T lymphocyte adhesion to epithelial cells through its binding to E-cadherin, which is selectively expressed on the basolateral side of epithelial cells [83]. The MHC-II^hi^/CD11c^hi^/CD11b^−^/CD103^+^ DC are also known as migratory DC. CD103^+^ DC can migrate to the draining lymph nodes (LN), produce IL-12 and are specialized in cross‑presentation [80,82]. MHC-II^hi^/CD11c^hi^/CD11b^+^/CD103^−^ DC are more efficient at presenting antigens on MHC II [84]. 

IKDC are characterized by the expression of CD11c^int^/Gr-1^+^/DX5^+^ or NK1.1^+^. They are present in the lung and express cell surface markers of DC, as well as NK cell markers [85,86]. IKDC could be considered as NK-like DC or DC-like NK cells, playing a major role as a distinct population of innate effectors against viral pathogens [85,87,88]. However, their classification [85,86,88,89,90], origin [91] and physiological roles [85,87,92,93] remain controversial. 

More recently, the TNF-α/inducible nitric oxide synthase (iNOS)-producing DC (tipDC) have been identified and found to control viral infections in the lung [94]. However, their role in hRSV or hMPV has not yet been described. TipDC, also known as inflammatory DC or activated macrophages, contribute to the control of the antimicrobial defense and are responsible for severe tissue damage in several models of infection [94,95,96,97]. However, based on the overlapping phenotypes of myeloid cells, further characterization of lung tipDC is needed in order to eliminate the possibility that they represent myeloid cells in a transient maturation stage, in response to infection. 

### 5.1. Lung DC Trafficking

Several studies have reported the trafficking and function of respiratory DC in response to hRSV or hMPV infection. Experimental evidence indicates that hRSV induces the recruitment of DC into the lungs and LN of BALB/c [98,99,100] and C57BL/6 [101] mice. In previous studies, I have observed that IKDC is the smallest DC subset recruited to the airways upon hRSV infection (two-fold) [98], followed by pDC (four-fold) and is the cDC the predominant DC population recruited to the lung after hRSV infection (20-fold) [98]. Some differences in the kinetics of pDC and IKDC into the lung of hMPV‑infected mice have been observed when compared to hRSV, as the recruitment of pDC and IKDC peaked by day eight after hMPV infection* versus* day three in hRSV-infected mice [98]. On the other hand, a similar trafficking pattern of cDC has been observed after hMPV infection when compared to hRSV [98], including a sustained recruitment of this cell population for about 18–21 days beyond the acute phase of infection [98,99]. It has also been found that CD103^+^ cDC are substantially decreased after hRSV infection [98,101], and the same effect has been observed in the lungs of hMPV-infected mice in which CD103^+^ cDC decreased even after three weeks of infection and returned to basal levels by week eight [98]. By contrast, after either hRSV or hMPV infection, CD11b^hi^ cDC increased about four-fold in the lung of infected mice [98,101]. Furthermore, hRSV infection stimulates the mobilization of both populations of cDC, as the numbers of CD103^+^ and CD11b^hi^ are increased in the lung-draining mediastinal LN [101].

### 5.2. Lung DC Activation

Upon viral challenge, lung cDC are activated and acquire a mature phenotype [23,26,28,102]. In that regard, previous experimental observations indicate that hRSV or hMPV induce the overexpression of surface molecules, including CD40, CD80, CD86, PD-L1, OX-40L and MHC-II, in pulmonary cDC as early as day one after infection and remained activated until three weeks after infection [98,101,103]. Moreover, the profile of cytokines produced by pulmonary pDC and cDC, infected with hRSV or hMPV, differ substantially. cDC produce IL-10, IL-1α, IL-6, CXCL1 and CCL11, while pulmonary pDC do not. However, both lung cDC and pDC produce IFN-α after hMPV infection* ex vivo*, while infection with hRSV did not stimulate the release of the antiviral cytokine [98]. hRSV also induces maturation of lung pDC, as indicated by the overexpression of CD80, CD86 and MHC class II molecules [104]. 

### 5.3. Lung pDC Function in hRSV Infection

It has been demonstrated that the balance between the numbers of pDC and cDC in the lung is important for the regulation of the immune response against hRSV. Smit* et al.* [105] demonstrated that when both pDC and cDC populations were expanded in hRSV-infected mice, that resulted in a decreased Th2 cell response, but an increased Th1 response and lower immunopathology. However, by depleting pDC and expanding cDC, the T-cell response was skewed towards Th2, resulting in an exacerbated inflammatory response. 

The specific role of pulmonary pDC in hRSV infection has also been explored by several other groups [100,104,106]. To this end, pDC have also been depleted with the monoclonal antibody (mAb), 120G8, that recognizes the murine surface antigen, CD317 (BST-2; mPDCA1), in several mouse strains [107]. Some studies have reported that the inflammatory response and the airway resistance was substantially exacerbated, and the lung viral titer was increased [100,104], suggesting that pDC play a protective role during hRSV infection. However, it is important to consider that, despite BST-2 antigen being expressed predominantly on pDC in naive mice, it is known that after viral infection or stimulation with type I or type II IFN, BST-2 is induced on most cell types [108]. Therefore, this fact should be taken into consideration for the interpretation of these studies. On the hand, it seems that pDC do not contribute significantly to the production of type I IFN* in vivo* in hRSV infection, as the levels of IFN‑α and IFN-β remained unchanged in hRSV-infected mice after successful depletion of pDC [106]. In fact, it has been reported that alveolar macrophages are the primary IFN-α producer in lung infections by RNA viruses [109]. In support to that, Pribul* et al.* [110] has demonstrated that alveolar macrophages significantly contribute to the production of IFN-α in hRSV infection. As for the role of DC subsets in hMPV infection, there are no reports exploring the contribution of these cells in the hMPV-induced immune response. Therefore, future experiments aimed to determine the role of DC subpopulations in hMPV-infected mice are needed to understand the contribution of these cells in hMPV-induced immune response. 

### 5.4. Impairment of Mouse Lung DC Response in hRSV and hMPV

Infection by hRSV and hMPV is characterized by short-lasting virus specific immunity and, often, long-term airway morbidity. Previous studies have revealed that hRSV or hMPV impair the capacity of human DC to present antigens to T-cells* in vitro* [33,101,111]. That detrimental effect has also been observed in experiments* in vivo* using the mouse model [98]. In that system, I have previously observed that, when compared with cDC from mock-infected mice, lung cDC from mice infected with hRSV or hMPV have an impaired capacity to present antigens to CD4^+^ T-cells that lasted beyond the acute phase of infection, suggesting that acute pneumovirus and metapneumovirus infections can alter the long-term immune function of pulmonary DC. Moreover, that inhibitory effect seems to be selective for lung cells, since that inhibitory effect was not observed when spleen cDC from the same infected mice were used [98]. The mechanisms by which hRSV and hMPV impair cDC function are largely unknown. However, one of the surface molecules that was upregulated after viral infection in lung cDC was programmed death-1 ligand (PD-L1) [98], which is known to inhibit some T-cell functions, including T-cell proliferation [112,113,114], suggesting that this molecule may play a role in the impaired capacity of cDC to present antigens to T-cells after hRSV and hMPV infection. 

The production of type I IFN and other cytokines by lung DC is also altered by hRSV and hMPV infection* in vivo* [98]. I have previously observed that lung pDC isolated from infected mice produced significantly lower levels of IFN-α, IL-6, TNF-α, CCL3, CCL4 and CCL5 in response to CpG ODN [98], indicating that both viruses are able to interfere with the capacity of pulmonary pDC to mount an antiviral response in response to a secondary stimulus. Age is also a factor that negatively impacts DC response in the lung. The recruitment of DC after hRSV infection can be impaired by the age of the infected individuals, as shown by Zhao* et al.* in aged mice (6–22 months-old) where the ability of lung DC to migrate to LN was compromised in hRSV-infected aged mice, with a decline in migration occurring as early as six months of age [115]. 

### 5.5. Contribution Lung DC in Vaccine Development

Data* in vivo* using the C57BL/6 mouse model of hRSV infection have indicated that after hRSV intranasal challenge to formalin-inactivated RSV (FI-RSV)-immunized mice, the numbers of CD11b^+^ and CD103^+^ cDC recruited into the lung are increased [116]. Considering that differences between these DC populations in priming the T-cell responses exist [80] and that the balance between the numbers of DC subsets influences the CD4^+^ Th1/Th2 responses in the lung [105], the development of hRSV and hMPV vaccines should consider the characterization of the lung DC subsets response and their contribution to prime an immune response against these viral infections, which will contribute toward the better design of an effective vaccine against these respiratory viral infections. 

## 6. Conclusions

Lung DC participate in the innate and adaptive immune response to hRSV and hMPV infections, indicating their critical role in the antiviral immunity to these paramyxoviruses. hRSV and hMPV can induce similar DC responses, as DC can be activated by both hRSV and hMPV infections* in vivo*. In addition, both viruses can induce the maturation and trafficking of the different DC populations from lung to LN. Moreover, they interfere with the T-cell response, as the antigen-presenting capacity of pulmonary DC to T-cells is impaired after hRSV or hMPV infection, which may contribute to the lack of protection and multiple reinfections by these viruses. On the other hand, hRSV and hMPV differentially induce the production of type I IFN in lung DC, as hMPV is a more potent inducer of the antiviral cytokine. 

Although many aspects of the immune mechanisms involving DC in hRSV infection have not been elucidated, considerable progress has been made with respect to our understanding of the role of pulmonary DC in hRSV infection. However, less is known regarding the interaction of hMPV with the lung DC, and in general, the mechanisms that regulate the host immune response to hMPV infection remain largely unknown. Additional studies are necessary to better understand the mechanisms that regulate the DC response in hRSV and hMPV infections.

## References

[1] Collins P.L., Crowe J., Knipe D.M., Howley P.M. (2007). Respiratory syncytial virus and metapneumovirus. Fileds Virology.

[2] Van den Hoogen B.G., de Jong J.C., Groen J., Kuiken T., de Groot R., Fouchier R.A., Osterhaus A.D. (2001). A newly discovered human pneumovirus isolated from young children with respiratory tract disease. Nat. Med..

[3] Domachowske J.B., Rosenberg H.F. (1999). Respiratory syncytial virus infection: Immune response, immunopathogenesis, and treatment. Clin. Microbiol. Rev..

[4] Easton A.J., Domachowske J.B., Rosenberg H.F. (2004). Animal pneumoviruses: Molecular genetics and pathogenesis. Clin. Microbiol. Rev..

[5] Blount R.E., Morris J.A., Savage R.E. (1956). Recovery of cytopathogenic agent from chimpanzees with coryza. Proc. Soc. Exp. Biol. Med..

[6] Boivin G., de Serres G., Cote S., Gilca R., Abed Y., Rochette L., Bergeron M.G., Dery P. (2003). Human metapneumovirus infections in hospitalized children. Emerg. Infect. Dis..

[7] Mullins J.A., Erdman D.D., Weinberg G.A., Edwards K., Hall C.B., Walker F.J., Iwane M., Anderson L.J. (2004). Human metapneumovirus infection among children hospitalized with acute respiratory illness. Emerg. Infect. Dis..

[8] Van den Hoogen B.G., van Doornum G.J., Fockens J.C., Cornelissen J.J., Beyer W.E., de Groot R., Osterhaus A.D., Fouchier R.A. (2003). Prevalence and clinical symptoms of human metapneumovirus infection in hospitalized patients. J. Infect. Dis..

[9] Williams J.V., Harris P.A., Tollefson S.J., Halburnt-Rush L.L., Pingsterhaus J.M., Edwards K.M., Wright P.F., Crowe J.E. (2004). Human metapneumovirus and lower respiratory tract disease in otherwise healthy infants and children. N. Engl. J. Med..

[10] Kahn J.S. (2006). Epidemiology of human metapneumovirus. Clin. Microbiol. Rev..

[11] Hall C.B., McCarthy C.A., Mandel G.L., Bennett J.E., Dolin R. (1995). Respiratory syncytial virus. Principles and Practice of Infectious Diseases.

[12] Hall C.B., Douglas R.G. (1981). Modes of transmission of respiratory syncytial virus. J. Pediatr..

[13] Lessler J., Reich N.G., Brookmeyer R., Perl T.M., Nelson K.E., Cummings D.A. (2009). Incubation periods of acute respiratory viral infections: A systematic review. Lancet Infect. Dis..

[14] Welliver R.C. (2003). Respiratory syncytial virus and other respiratory viruses. Pediatr. Infect. Dis. J..

[15] Hermos C.R., Vargas S.O., McAdam A.J. (2010). Human metapneumovirus. Clin. Lab. Med..

[16] Papenburg J., Hamelin M.E., Ouhoummane N., Carbonneau J., Ouakki M., Raymond F., Robitaille L., Corbeil J., Caouette G., Frenette L. (2012). Comparison of risk factors for human metapneumovirus and respiratory syncytial virus disease severity in young children. J. Infect. Dis..

[17] Papenburg J., Boivin G. (2010). The distinguishing features of human metapneumovirus and respiratory syncytial virus. Rev. Med. Virol..

[18] Van Drunen Littel-van den Hurk S., Watkiss E.R. (2012). Pathogenesis of respiratory syncytial virus. Curr. Opin. Virol..

[19] Pavlin J.A., Hickey A.C., Ulbrandt N., Chan Y.P., Endy T.P., Boukhvalova M.S., Chunsuttiwat S., Nisalak A., Libraty D.H., Green S. (2008). Human metapneumovirus reinfection among children in Thailand determined by ELISA using purified soluble fusion protein. J. Infect. Dis..

[20] Ebihara T., Endo R., Ishiguro N., Nakayama T., Sawada H., Kikuta H. (2004). Early reinfection with human metapneumovirus in an infant. J. Clin. Microbiol..

[21] Ohuma E.O., Okiro E.A., Ochola R., Sande C.J., Cane P.A., Medley G.F., Bottomley C., Nokes D.J. (2012). The natural history of respiratory syncytial virus in a birth cohort: The influence of age and previous infection on reinfection and disease. Am. J. Epidemiol..

[22] Graham B.S. (2011). Biological challenges and technological opportunities for respiratory syncytial virus vaccine development. Immunol. Rev..

[23] Steinman R.M. (2012). Decisions about dendritic cells: Past, present, and future. Annu. Rev. Immunol..

[24] Grayson M.H., Holtzman M.J. (2007). Emerging role of dendritic cells in respiratory viral infection. J. Mol. Med..

[25] Reis e Sousa C. (2004). Activation of dendritic cells: Translating innate into adaptive immunity. Curr. Opin. Immunol..

[26] Banchereau J., Briere F., Caux C., Davoust J., Lebecque S., Liu Y.T., Pulendran B., Palucka K. (2000). Immunobiology of dendritic cells. Ann. Rev. Immunol..

[27] Pulendran B., Palucka K., Banchereau J. (2001). Sensing pathogens and tuning immune responses. Science.

[28] Steinman R.M., Cohn Z.A. (1973). Identification of a novel cell type in peripheral lymphoid organs of mice. I. Morphology, quantitation, tissue distribution. J. Exp. Med..

[29] Stumbles P.A., Upham J.W., Holt P.G. (2003). Airway dendritic cells: Co-ordinators of immunological homeostasis and immunity in the respiratory tract. APMIS.

[30] Schon-Hegrad M.A., Oliver J., McMenamin P.G., Holt P.G. (1991). Studies on the density, distribution, and surface phenotype of intraepithelial class II major histocompatibility complex antigen (Ia)-bearing dendritic cells (DC) in the conducting airways. J. Exp. Med..

[31] McWilliam A.S., Napoli S., Marsh A.M., Pemper F.L., Nelson D.J., Pimm C.L., Stumbles P.A., Wells T.N., Holt P.G. (1996). Dendritic cells are recruited into the airway epithelium during the inflammatory response to a broad spectrum of stimuli. J. Exp. Med..

[32] Manicassamy S., Pulendran B. (2011). Dendritic cell control of tolerogenic responses. Immunol. Rev..

[33] Guerrero-Plata A., Casola A., Suarez G., Yu X., Spetch L., Peeples M.E., Garofalo R.P. (2006). Differential response of dendritic cells to human metapneumovirus and respiratory syncytial virus. Am. J. Respir. Cell Mol. Biol..

[34] Le Nouen C., Munir S., Losq S., Winter C.C., McCarty T., Stephany D.A., Holmes K.L., Bukreyev A., Rabin R.L., Collins P.L. (2009). Infection and maturation of monocyte-derived human dendritic cells by human respiratory syncytial virus, human metapneumovirus, and human parainfluenza virus type 3. Virology.

[35] Jones A., Morton I., Hobson L., Evans G.S., Everard M.L. (2006). Differentiation and immune function of human dendritic cells following infection by respiratory syncytial virus. Clin. Exp. Immunol..

[36] De Graaff P.M., de Jong E.C., van Capel T.M., van Dijk M.E., Roholl P.J., Boes J., Luytjes W., Kimpen J.L., van Bleek G.M. (2005). Respiratory syncytial virus infection of monocyte-derived dendritic cells decreases their capacity to activate CD4 T cells. J. Immunol..

[37] Bartz H., Turkel O., Hoffjan S., Rothoeft T., Gonschorek A., Schauer U. (2003). Respiratory syncytial virus decreases the capacity of myeloid dendritic cells to induce interferon-gamma in naive T cells. Immunology.

[38] Hornung V., Schlender J., Guenthner-Biller M., Rothenfusser S., Endres S., Conzelmann K.K., Hartmann G. (2004). Replication-dependent potent IFN-alpha induction in human plasmacytoid dendritic cells by a single-stranded RNA virus. J. Immunol..

[39] Johnson T.R., Johnson C.N., Corbett K.S., Edwards G.C., Graham B.S. (2011). Primary human mDC1, mDC2, and pDC dendritic cells are differentially infected and activated by respiratory syncytial virus. PLoS One.

[40] Gonzalez P.A., Prado C.E., Leiva E.D., Carreno L.J., Bueno S.M., Riedel C.A., Kalergis A.M. (2008). Respiratory syncytial virus impairs T cell activation by preventing synapse assembly with dendritic cells. Proc. Natl. Acad. Sci. USA.

[41] Bartz H., Buning-Pfaue F., Turkel O., Schauer U. (2002). Respiratory syncytial virus induces prostaglandin E2, IL-10 and IL-11 generation in antigen presenting cells. Clin. Exp. Immunol..

[42] Rudd B.D., Luker G.D., Luker K.E., Peebles R.S., Lukacs N.W. (2007). Type I interferon regulates respiratory virus infected dendritic cell maturation and cytokine production. Viral Immunol..

[43] Munir S., Le N.C., Luongo C., Buchholz U.J., Collins P.L., Bukreyev A. (2008). Nonstructural proteins 1 and 2 of respiratory syncytial virus suppress maturation of human dendritic cells. J. Virol..

[44] Johnson T.R., McLellan J.S., Graham B.S. (2012). Respiratory syncytial virus glycoprotein G interacts with DC-SIGN and L-SIGN to activate ERK1 and ERK2. J. Virol..

[45] Morris S., Swanson M.S., Lieberman A., Reed M., Yue Z., Lindell D.M., Lukacs N.W. (2011). Autophagy-mediated dendritic cell activation is essential for innate cytokine production and APC function with respiratory syncytial virus responses. J. Immunol..

[46] Kundu M., Thompson C.B. (2008). Autophagy: Basic principles and relevance to disease. Ann. Rev. Pathol..

[47] Ank N., Paludan S.R. (2009). Type III IFNs: New layers of complexity in innate antiviral immunity. Biofactors.

[48] Piehler J., Thomas C., Garcia K.C., Schreiber G. (2012). Structural and dynamic determinants of type I interferon receptor assembly and their functional interpretation. Immunol. Rev..

[49] Garcia-Sastre A., Biron C.A. (2006). Type 1 interferons and the virus-host relationship: A lesson in detente. Science.

[50] Takeuchi O., Akira S. (2008). MDA5/RIG-I and virus recognition. Curr. Opin. Immunol..

[51] Baum A., Garcia-Sastre A. (2009). Induction of type I interferon by RNA viruses: Cellular receptors and their substrates. Amino Acids.

[52] Schlender J., Hornung V., Finke S., Gunthner-Biller M., Marozin S., Brzozka K., Moghim S., Endres S., Hartmann G., Conzelmann K.K. (2005). Inhibition of toll-like receptor 7- and 9-mediated alpha/beta interferon production in human plasmacytoid dendritic cells by respiratory syncytial virus and measles virus. J. Virol..

[53] Castro S.M., Chakraborty K., Guerrero-Plata A. (2011). Cigarette smoke suppresses TLR-7 stimulation in response to virus infection in plasmacytoid dendritic cells. Toxicol. Vitro.

[54] Goutagny N., Jiang Z., Tian J., Parroche P., Schickli J., Monks B.G., Ulbrandt N., Ji H., Kiener P.A., Coyle A.J. (2010). Cell type-specific recognition of human metapneumoviruses (HMPVs) by retinoic acid-inducible gene I (RIG-I) and TLR7 and viral interference of RIG-I ligand recognition by HMPV-B1 phosphoprotein. J. Immunol..

[55] Banos-Lara Mdel R., Ghosh A., Guerrero-Plata A. (2013). Critical role of MDA5 in the interferon response induced by human metapneumovirus infection in dendritic cells and* in vivo*. J. Virol..

[56] Kolli D., Bao X., Liu T., Hong C., Wang T., Garofalo R.P., Casola A. (2011). Human metapneumovirus glycoprotein G inhibits TLR4-dependent signaling in monocyte-derived dendritic cells. J. Immunol..

[57] Shingai M., Azuma M., Ebihara T., Sasai M., Funami K., Ayata M., Ogura H., Tsutsumi H., Matsumoto M., Seya T. (2008). Soluble G protein of respiratory syncytial virus inhibits Toll-like receptor 3/4-mediated IFN-beta induction. Int. Immunol..

[58] Henderson F.W., Collier A.M., Clyde W.A., Denny F.W. (1979). Respiratory-syncytial-virus infections, reinfections and immunity. A prospective, longitudinal study in young children. New Engl. J. Med..

[59] Falsey A.R., Walsh E.E. (2006). Viral pneumonia in older adults. Clin. Infect. Dis..

[60] Hall C.B., Walsh E.E., Long C.E., Schnabel K.C. (1991). Immunity to and frequency of reinfection with respiratory syncytial virus. J. Infect. Dis..

[61] Rothoeft T., Fischer K., Zawatzki S., Schulz V., Schauer U., Korner Rettberg C. (2007). Differential response of human naive and memory/effector T cells to dendritic cells infected by respiratory syncytial virus. Clin. Exp. Immunol..

[62] Cespedes P.F., Gonzalez P.A., Kalergis A.M. (2013). Human metapneumovirus keeps dendritic cells from priming antigen-specific naive T cells. Immunology.

[63] Le Nouen C., Hillyer P., Munir S., Winter C.C., McCarty T., Bukreyev A., Collins P.L., Rabin R.L., Buchholz U.J. (2010). Effects of human respiratory syncytial virus, metapneumovirus, parainfluenza virus 3 and influenza virus on CD4+ T cell activation by dendritic cells. PLoS One.

[64] Demedts I.K., Brusselle G.G., Vermaelen K.Y., Pauwels R.A. (2005). Identification and characterization of human pulmonary dendritic cells. Am. J. Respir. Cell Mol. Biol..

[65] Masten B.J., Olson G.K., Tarleton C.A., Rund C., Schuyler M., Mehran R., Archibeque T., Lipscomb M.F. (2006). Characterization of myeloid and plasmacytoid dendritic cells in human lung. J. Immunol..

[66] Condon T.V., Sawyer R.T., Fenton M.J., Riches D.W. (2011). Lung dendritic cells at the innate-adaptive immune interface. J. Leukoc. Biol..

[67] Bratke K., Lommatzsch M., Julius P., Kuepper M., Kleine H.D., Luttmann W., Christian Virchow J. (2007). Dendritic cell subsets in human bronchoalveolar lavage fluid after segmental allergen challenge. Thorax.

[68] Lommatzsch M., Bratke K., Bier A., Julius P., Kuepper M., Luttmann W., Virchow J.C. (2007). Airway dendritic cell phenotypes in inflammatory diseases of the human lung. Eur. Respir. J..

[69] Gill M.A., Palucka A.K., Barton T., Ghaffar F., Jafri H., Banchereau J., Ramilo O. (2005). Mobilization of plasmacytoid and myeloid dendritic cells to mucosal sites in children with respiratory syncytial virus and other viral respiratory infections. J. Infect. Dis..

[70] Gill M.A., Long K., Kwon T., Muniz L., Mejias A., Connolly J., Roy L., Banchereau J., Ramilo O. (2008). Differential recruitment of dendritic cells and monocytes to respiratory mucosal sites in children with influenza virus or respiratory syncytial virus infection. J. Infect. Dis..

[71] Silver E., Yin-DeClue H., Schechtman K.B., Grayson M.H., Bacharier L.B., Castro M. (2009). Lower levels of plasmacytoid dendritic cells in peripheral blood are associated with a diagnosis of asthma 6 yr after severe respiratory syncytial virus bronchiolitis. Pediatr. Allergy Immunol..

[72] Gilliet M., Cao W., Liu Y.J. (2008). Plasmacytoid dendritic cells: Sensing nucleic acids in viral infection and autoimmune diseases. Nat. Rev. Immunol..

[73] Honda K., Yanai H., Takaoka A., Taniguchi T. (2005). Regulation of the type I IFN induction: A current view. Int. Immunol..

[74] Colonna M., Trinchieri G., Liu Y.J. (2004). Plasmacytoid dendritic cells in immunity. Nat. Immunol..

[75] Swiecki M., Colonna M. (2010). Unraveling the functions of plasmacytoid dendritic cells during viral infections, autoimmunity, and tolerance. Immunol. Rev..

[76] Young L.J., Wilson N.S., Schnorrer P., Proietto A., ten Broeke T., Matsuki Y., Mount A.M., Belz G.T., O'Keeffe M., Ohmura-Hoshino M. (2008). Differential MHC class II synthesis and ubiquitination confers distinct antigen-presenting properties on conventional and plasmacytoid dendritic cells. Nat. Immunol..

[77] Sozzani S., Vermi W., Del Prete A., Facchetti F. (2010). Trafficking properties of plasmacytoid dendritic cells in health and disease. Trends Immunol..

[78] Villadangos J.A., Young L. (2008). Antigen-presentation properties of plasmacytoid dendritic cells. Immunity.

[79] Lambrecht B.N., Hammad H. (2009). Biology of lung dendritic cells at the origin of asthma. Immunity.

[80] del Rio M.L., Rodriguez-Barbosa J.I., Kremmer E., Forster R. (2007). CD103- and CD103+ bronchial lymph node dendritic cells are specialized in presenting and cross-presenting innocuous antigen to CD4+ and CD8+ T cells. J. Immunol..

[81] Coombes J.L., Siddiqui K.R., Arancibia-Carcamo C.V., Hall J., Sun C.M., Belkaid Y., Powrie F. (2007). A functionally specialized population of mucosal CD103+ DCs induces Foxp3+ regulatory T cells via a TGF-beta and retinoic acid-dependent mechanism. J. Exp. Med..

[82] del Rio M.L., Bernhardt G., Rodriguez-Barbosa J.I., Forster R. (2010). Development and functional specialization of CD103+ dendritic cells. Immunol. Rev..

[83] Cepek K.L., Shaw S.K., Parker C.M., Russell G.J., Morrow J.S., Rimm D.L., Brenner M.B. (1994). Adhesion between epithelial cells and T lymphocytes mediated by E-cadherin and the alpha E beta 7 integrin. Nature.

[84] Dudziak D., Kamphorst A.O., Heidkamp G.F., Buchholz V.R., Trumpfheller C., Yamazaki S., Cheong C., Liu K., Lee H.W., Park C.G. (2007). Differential antigen processing by dendritic cell subsets *in vivo*. Science.

[85] Chan C.W., Crafton E., Fan H.N., Flook J., Yoshimura K., Skarica M., Brockstedt D., Dubensky T.W., Stins M.F., Lanier L.L. (2006). Interferon-producing killer dendritic cells provide a link between innate and adaptive immunity. Nat. Med..

[86] Taieb J., Chaput N., Menard C., Apetoh L., Ullrich E., Bonmort M., Pequignot M., Casares N., Terme M., Flament C. (2006). A novel dendritic cell subset involved in tumor immunosurveillance. Nat. Med..

[87] Bonmort M., Dalod M., Mignot G., Ullrich E., Chaput N., Zitvogel L. (2008). Killer dendritic cells: IKDC and the others. Curr. Opin. Immunol..

[88] Chauvin C., Josien R. (2008). Dendritic cells as killers: Mechanistic aspects and potential roles. J. Immunol..

[89] Blasius A.L., Barchet W., Cella M., Colonna M. (2007). Development and function of murine B220+CD11c+NK1.1+ cells identify them as a subset of NK cells. J. Exp. Med..

[90] Vosshenrich C.A., Lesjean-Pottier S., Hasan M., Richard-Le Goff O., Corcuff E., Mandelboim O., di Santo J.P. (2007). CD11cloB220+ interferon-producing killer dendritic cells are activated natural killer cells. J. Exp. Med..

[91] Welner R.S., Pelayo R., Garrett K.P., Chen X., Perry S.S., Sun X.H., Kee B.L., Kincade P.W. (2007). Interferon-producing killer dendritic cells (IKDCs) arise via a unique differentiation pathway from primitive c-kitHiCD62L+ lymphoid progenitors. Blood.

[92] Caminschi I., Ahmet F., Heger K., Brady J., Nutt S.L., Vremec D., Pietersz S., Lahoud M.H., Schofield L., Hansen D.S. (2007). Putative IKDCs are functionally and developmentally similar to natural killer cells, but not to dendritic cells. J. Exp. Med..

[93] Spits H., Lanier L.L. (2007). Natural killer or dendritic: What’s in a name?. Immunity.

[94] Aldridge J.R., Moseley C.E., Boltz D.A., Negovetich N.J., Reynolds C., Franks J., Brown S.A., Doherty P.C., Webster R.G., Thomas P.G. (2009). TNF/iNOS-producing dendritic cells are the necessary evil of lethal influenza virus infection. Proc. Natl. Acad. Sci. USA.

[95] Serbina N.V., Salazar-Mather T.P., Biron C.A., Kuziel W.A., Pamer E.G. (2003). TNF/iNOS-producing dendritic cells mediate innate immune defense against bacterial infection. Immunity.

[96] Bosschaerts T., Guilliams M., Stijlemans B., Morias Y., Engel D., Tacke F., Herin M., de Baetselier P., Beschin A. (2010). Tip-DC development during parasitic infection is regulated by IL-10 and requires CCL2/CCR2, IFN-gamma and MyD88 signaling. PLoS Pathog..

[97] Hespel C., Moser M. (2012). Role of inflammatory dendritic cells in innate and adaptive immunity. Eur. J. Immunol..

[98] Guerrero-Plata A., Kolli D., Hong C., Casola A., Garofalo R.P. (2009). Subversion of pulmonary dendritic cell function by paramyxovirus infections. J. Immunol..

[99] Beyer M., Bartz H., Horner K., Doths S., Koerner-Rettberg C., Schwarze J. (2004). Sustained increases in numbers of pulmonary dendritic cells after respiratory syncytial virus infection. J. Allergy Clin. Immunol..

[100] Smit J.J., Rudd B.D., Lukacs N.W. (2006). Plasmacytoid dendritic cells inhibit pulmonary immunopathology and promote clearance of respiratory syncytial virus. J. Exp. Med..

[101] Lukens M.V., Kruijsen D., Coenjaerts F.E., Kimpen J.L., van Bleek G.M. (2009). Respiratory syncytial virus-induced activation and migration of respiratory dendritic cells and subsequent antigen presentation in the lung-draining lymph node. J. Virol..

[102] Mellman I., Steinman R.M. (2001). Dendritic cells: Specialized and regulated antigen processing machines. Cell.

[103] Wythe S.E., Dodd J.S., Openshaw P.J., Schwarze J. (2012). OX40 ligand and programmed cell death 1 ligand 2 expression on inflammatory dendritic cells regulates CD4 T cell cytokine production in the lung during viral disease. J. Immunol..

[104] Wang H., Peters N., Schwarze J. (2006). Plasmacytoid dendritic cells limit viral replication, pulmonary inflammation, and airway hyperresponsiveness in respiratory syncytial virus infection. J. Immunol..

[105] Smit J.J., Lindell D.M., Boon L., Kool M., Lambrecht B.N., Lukacs N.W. (2008). The balance between plasmacytoid DC *versus* conventional DC determines pulmonary immunity to virus infections. PLoS One.

[106] Jewell N.A., Vaghefi N., Mertz S.E., Akter P., Peebles R.S., Bakaletz L.O., Durbin R.K., Flano E., Durbin J.E. (2007). Differential type I interferon induction by respiratory syncytial virus and influenza a virus *in vivo*. J. Virol..

[107] Asselin-Paturel C., Brizard G., Pin J.J., Briere F., Trinchieri G. (2003). Mouse strain differences in plasmacytoid dendritic cell frequency and function revealed by a novel monoclonal antibody. J. Immunol..

[108] Blasius A.L., Giurisato E., Cella M., Schreiber R.D., Shaw A.S., Colonna M. (2006). Bone marrow stromal cell antigen 2 is a specific marker of type I IFN-producing cells in the naive mouse, but a promiscuous cell surface antigen following IFN stimulation. J. Immunol..

[109] Kumagai Y., Takeuchi O., Kato H., Kumar H., Matsui K., Morii E., Aozasa K., Kawai T., Akira S. (2007). Alveolar macrophages are the primary interferon-alpha producer in pulmonary infection with RNA viruses. Immunity.

[110] Pribul P.K., Harker J., Wang B., Wang H., Tregoning J.S., Schwarze J., Openshaw P.J. (2008). Alveolar macrophages are a major determinant of early responses to viral lung infection but do not influence subsequent disease development. J. Virol..

[111] Biacchesi S., Skiadopoulos M.H., Boivin G., Hanson C.T., Murphy B.R., Collins P.L., Buchholz U.J. (2003). Genetic diversity between human metapneumovirus subgroups. Virology.

[112] Erickson J.J., Gilchuk P., Hastings A.K., Tollefson S.J., Johnson M., Downing M.B., Boyd K.L., Johnson J.E., Kim A.S., Joyce S. (2012). Viral acute lower respiratory infections impair CD8+ T cells through PD-1. J. Clin. Invest..

[113] Carter L., Fouser L.A., Jussif J., Fitz L., Deng B., Wood C.R., Collins M., Honjo T., Freeman G.J., Carreno B.M. (2002). PD-1:PD-L inhibitory pathway affects both CD4(+) and CD8(+) T cells and is overcome by IL-2. Eur. J. Immunol..

[114] Freeman G.J., Long A.J., Iwai Y., Bourque K., Chernova T., Nishimura H., Fitz L.J., Malenkovich N., Okazaki T., Byrne M.C. (2000). Engagement of the PD-1 immunoinhibitory receptor by a novel B7 family member leads to negative regulation of lymphocyte activation. J. Exp. Med..

[115] Zhao J., Legge K., Perlman S. (2011). Age-related increases in PGD(2) expression impair respiratory DC migration, resulting in diminished T cell responses upon respiratory virus infection in mice. J. Clin. Invest..

[116] Kruijsen D., Schijf M.A., Lukens M.V., van Uden N.O., Kimpen J.L., Coenjaerts F.E., van Bleek G.M. (2011). Local innate and adaptive immune responses regulate inflammatory cell influx into the lungs after vaccination with formalin inactivated RSV. Vaccine.

